# Microscopic origin of the spatial and temporal precision in biological systems

**DOI:** 10.1016/j.bpr.2025.100197

**Published:** 2025-01-28

**Authors:** Anupam Mondal, Anatoly B. Kolomeisky

**Affiliations:** 1 Center for Theoretical Biological Physics, Rice University, Houston, Texas; 2 Department of Chemistry, Rice University, Houston, Texas; 3 Department of Chemical and Biomolecular Engineering, Rice University, Houston, Texas

## Abstract

All living systems display remarkable spatial and temporal precision, despite operating in intrinsically fluctuating environments. It is even more surprising given that biological phenomena are regulated by multiple chemical reactions that are also random. Although the underlying molecular mechanisms of surprisingly high precision in biology remain not well understood, a novel theoretical picture that relies on the coupling of relevant stochastic processes has recently been proposed and applied to explain different phenomena. To illustrate this approach, in this review, we discuss two systems that exhibit precision control: spatial regulation in bacterial cell size and temporal regulation in the timing of cell lysis by *λ* bacteriophage. In cell-size regulation, it is argued that a balance between stochastic cell growth and cell division processes leads to a narrow distribution of cell sizes. In cell lysis, it is shown that precise timing is due to the coupling of holin protein accumulation and the breakage of the cellular membrane. The stochastic coupling framework also allows us to explicitly evaluate dynamic properties for both biological systems, eliminating the need to utilize the phenomenological concept of thresholds. Excellent agreement with experimental observations is observed, supporting the proposed theoretical ideas. These observations also suggest that the stochastic coupling method captures the important aspects of molecular mechanisms of precise cellular regulation, providing a powerful new tool for more advanced investigations of complex biological phenomena.

## Why it matters

In this review, we provided novel microscopic insights into how biological systems achieve precise control over spatial and temporal processes despite the inherent randomness of molecular interactions. By examining bacterial cell-size regulation and the timing of cell lysis in *λ* bacteriophage, we demonstrate that cells can harness stochastic processes to ensure reliable outcomes. In bacterial cells, balanced growth and division rates lead to stable size distributions, whereas, in *λ* phage, holin protein accumulation triggers timely lysis, achieving effective threshold-like behavior. These studies highlight universal mechanisms by which cells adapt precision strategies in size and timing, advancing our understanding of cellular regulation. It has potential applications in synthetic biology, where predictable control over biological functions is crucial.

## Introduction

One of the most outstanding features of all biological systems is the precision in their functioning when finely tuned processes enable living organisms to maintain homeostasis, respond to environmental cues, and propagate life across generations (1,2). However, this precision emerges amid inherent stochasticity, with molecular interactions and cellular events often governed by probabilistic rules rather than deterministic certainty (3). The fact that multiple chemical processes regulate biological processes makes these observations even more surprising since all chemical reactions are random. Numerous studies have investigated the origin of precision in biology, concentrating on how living systems achieve consistent outcomes despite intrinsic noise, fluctuations in molecular interactions, and variations in external conditions (4,5). Understanding the molecular mechanisms that lead to precision is not only crucial for deciphering fundamental biological principles but also for applications in synthetic biology, biomedicine, and bioengineering, where emulating or manipulating precision can lead to innovative therapeutic strategies and biological devices (6).

In random fluctuating environments, cells have evolved diverse regulatory strategies to ensure that essential processes occur with remarkable accuracy. One approach is the tight coordination of spatial dimensions, supporting the observations that components within a cell are organized to optimize functionality and maintain structural integrity (7,8,9,10). Another approach involves temporal regulation, where cells time their responses to environmental signals or internal cues to achieve the necessary fidelity in cellular dynamics (11,12,13,14,15,16). These dual strategies, spatial and temporal precision, are particularly evident in cellular processes that require high reliability for organism survival and function, such as cell-size regulation and timing of cell lysis by bacteriophages. By investigating these mechanisms, one can gain crucial insights into how precision arises within a framework governed by molecular randomness.

Spatial precision is crucial for the proper organization and functioning of cellular components. One well-studied example is the regulation of cell size in bacteria, which ensures that cells grow to an optimal size before division. In many organisms, cells employ feedback mechanisms to sense and adjust their growth, maintaining a size that balances metabolic demands with structural stability. For instance, in *Escherichia coli* and *Saccharomyces cerevisiae*, cell size is modulated by a balance between growth rates and division frequency, with mechanisms in place to ensure that cells do not grow too large or remain too small, which would jeopardize their efficiency and functionality (17,18,19,20). In bacteria, it has been proposed that this process probably follows one of two limiting phenomenological behaviors, known as “sizer” or “adder” models (17,21). In the sizer picture, the originally small cells add more volume, whereas the originally large cells add smaller volume, ensuring that, after several divisions, cell sizes reach some stationary limit. In the adder picture, cells add the same volume between divisions, also leading to the constant stationary size of the bacterial cells. These processes highlight the remarkable precision in spatial regulation within cells and reveal the importance of maintaining physical constraints for optimal cellular performance.

Temporal precision is equally critical, as it ensures that cellular events occur at the right time to maximize efficiency and adaptability. One striking example is the timing of cell lysis in *λ* bacteriophage infection, where precise timing determines the success of viral propagation (13,22,23,24). After infecting a bacterium, the *λ*-phage controls the expression of proteins that regulate lysis, ensuring that the host cell ruptures only after the viral genome has replicated sufficiently. This timing maximizes viral particle production before cell lysis releases the phage progeny into the surrounding environment. The *λ*-phage virus thus demonstrates a high degree of temporal control over cellular processes, coordinating genetic circuits and protein synthesis in a manner that allows for optimal timing in the face of environmental and biological noise.

The molecular mechanisms that lead to high precision in biological systems remain not well understood, although various ideas have been proposed, including feedback regulation, noise cancellation, and energy dissipation (4,5). Recently, a new theoretical framework has been proposed to clarify the microscopic origin of precision in living organisms (25,26). It argues that the coupling of several stochastic processes might lead to effectively deterministic dynamics and spatial outcomes. This is a powerful approach since it eliminates the need to use phenomenological concepts such as thresholds, providing a simple and physically appealing explanation of high precision in biology. To illustrate this method, this review focuses on the mechanisms and principles underlying spatial and temporal precision in specific biological systems, such as cell-size regulation and *λ* bacteriophage cell lysis as representative examples. We analyze these two processes in detail to elucidate how biological systems achieve precision in inherently random environments. Understanding these mechanisms deepens our knowledge of fundamental biological processes. It also provides valuable insights into potential applications, from developing precision-targeted therapies to engineering synthetic biological systems that mimic or control biological precision.

## Spatial precision in bacterial cell size regulation

**Figure 1:**
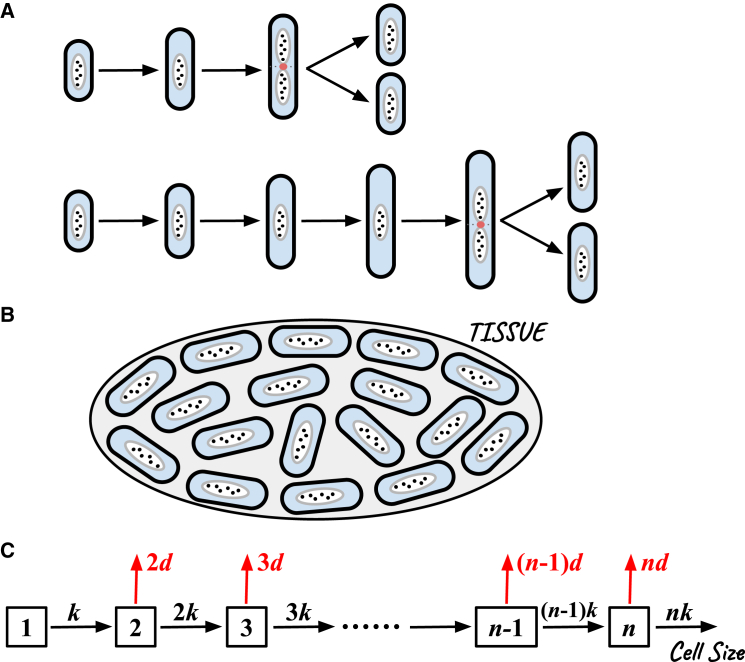
Stochastic and deterministic aspects of cell size regulation (*A*) Schematic representation of cell growth and division as stochastic processes. Individual cells undergo growth followed by division events, each introducing variability in the size of daughter cells. (*B*) After division, newly formed cells remain within the structural organization of the originating tissue, illustrating that cells tend to maintain a consistent size within a narrow distribution, supporting deterministic-like regulation of cell sizes. (*C*) Discrete-state stochastic model of cell-size regulation in bacteria. Cell length is represented by the discrete variable *n*, corresponding to the number of growth and division-associated proteins. Growth occurs at rate kn, proportional to cell length, and division occurs at rate dn. This coupling allows the division to preferentially occur within a narrow range of cell sizes, reducing the need for specific threshold assumptions.

Cell growth and division in bacteria is one of the most striking examples of spatial precision in biology, particularly due to the ability to produce cells of nearly uniform sizes despite the underlying stochastic nature of these processes. As depicted schematically in Fig. 1 *A*, bacterial growth and division are inherently stochastic events, yet these processes result in daughter cells that remain structurally similar and reach the same sizes as parent cells. After division, the newly formed cells retain their spatial organization within the tissue from which they originated (Fig. 1 *B*). Although there is considerable variation in cell shapes and sizes across different bacterial species, individual species tend to have a narrow distribution of cell sizes, maintaining remarkable reproducibility (17,18,27,28,29). This uniformity is thought to reflect a state of homeostasis, a dynamic “equilibrium” where biological systems optimize function by keeping physiological parameters as stable as possible. In bacteria, this includes not only molecular compositions but also the shapes and sizes of cells, which are carefully regulated to support optimal cellular performance. It is known that deviations from cell size uniformity might indicate serious deceases such as cancer (20).

The microscopic mechanisms that lead to tight regulation of cell sizes remain not well clarified (17,18,21,28,30). To explain spatial precision, the concept of cell cycle checkpoints, which assumes that there are devoted biochemical pathways that monitor the major cell cycle events, has been proposed (31). However, experimental observations indicate that bacteria lack such checkpoints because of the possible overlap of DNA replication and cell division machinery (21,27). At the same time, bacterial cells still exhibit a narrow distribution of their sizes and shapes (17), apparently utilizing different mechanisms.

Two dominant phenomenological approaches, called sizer and adder models, have been proposed to explain how the uniformity in cell-size distribution can be achieved (17,21). The sizer model posits that cells reach a target size through a feedback mechanism that curbs the growth of large cells while promoting growth in smaller ones, ultimately bringing cell sizes closer to a characteristic average size. In contrast, the adder model suggests that cells add a fixed amount of volume or length before dividing, irrespective of their initial size. In this model, large cells incrementally shrink in size over divisions, whereas smaller cells gradually grow larger, both eventually converging around an optimal size (17). These principles can rationalize why cells of a specific tissue can produce similar sizes despite individual variations in growth conditions and rates. However, these methods are not able to explain microscopically why the size distributions are so narrow and why cells might choose one of those two strategies.

Significant progress in our understanding of mechanisms of cell-size regulation in bacteria came with recent experimental advances, particularly in single-cell microfluidics, that allowed researchers to quantify the dynamics of cell growth and division with a single-cell precision (17,28). These experiments provided quantitative support for the adder model in various bacterial species (17,28). Based on these observations it was proposed that there are two necessary conditions for the bacterial cell-size regulation to occur with such precision, namely balanced biosynthesis and threshold accumulation (18,28,30,32).

The first condition, balanced biosynthesis, means that cells maintain proportionality between protein production and cell volume, ensuring that the number of growth-related protein molecules increases in tandem with cell size. In addition, this condition also implies that these proteins are synthesized on timescales that are much faster than timescales for cell growth and division. This can be viewed as a kind of “adiabatic approximation” according to which the most relevant molecular players are assembled much faster than the dynamics of cell growth and division. This condition seems to be realistic because the times to synthesize proteins are known to be ∼1 ms, whereas it takes minutes and hours for cells to grow and divide.

The second condition states that, simultaneously, cells must accumulate division-related molecules up to some threshold, which triggers the division only when the number of those molecules reaches sufficient levels. Although this picture provides a useful phenomenological framework, the specific microscopic mechanisms that produce these thresholds remain elusive. This is the weakest point of current theoretical understanding of cell-size regulation since the thresholds must be the result of several biochemical processes (17,25). Postulating the existence of thresholds does not clarify the underlying molecular picture of high spatial precision in biological systems.

To resolve these contradictions, a novel stochastic-coupling hypothesis of cell-size regulation has been proposed recently (25). According to this approach, several stochastic processes can be coupled to produce effectively almost deterministic outcomes of narrow cell-size distributions. To test this idea, a stochastic model of cell-size regulation, as illustrated in Fig. 1 *C*, has been introduced (25). In this model, cell growth is described as a quasi-one-dimensional process, which reflects the rod-like structure of bacterial cells such as *E. coli* and *Bacillus subtilis*. The increase in cell length is associated with changes in protein numbers related to cell elongation and division, notably involving proteins like FtsZ, a major player in bacterial cell constriction during division (33,34,35,36,37,38). In this model, the cell length is quantified as a discrete variable *n*, which represents the number of growth- or division-associated proteins (25). Since the relevant proteins are created much faster than cell growth and division, it is reasonable to follow only a single discrete variable because the ratio between them is close to being constant all the time. This is reasonable for the conditions when the nutrients are sufficiently available. It assumed that the growth is a stochastic process that occurs with a rate proportional to the current cell length *n*, specifically kn, where *k* is the growth rate constant. Similarly, it is assumed that the division can be described by another stochastic process occurring with a rate dn, where *d* is the division rate constant (25). This minimal theoretical model introduces stochasticity by coupling cell growth and division and treating them as effective stochastic processes (akin to chemical reactions), allowing for variability while still producing a tightly regulated range of cell sizes.

The important advantage of this theoretical method is that the stochastic coupling eliminates the need for predefined thresholds, sidestepping a major limitation of deterministic approaches. The two stochastic processes, growth and division, are coupled because the corresponding rates are assumed to be proportional to the current length of the cell, *n*. This allows us to account for stochasticity and simultaneously explains why the division is preferably taking place at some narrow range of cell sizes. This is because, in this range, the growth and division rates are comparable. Furthermore, the model can be explicitly solved, allowing for a better understanding of the physics of tight cell-size regulation.

The stochastic framework can be successfully utilized to explain why the cell-size regulation follows the adder scenario when the same length is added between every division. To understand this, let us consider a single cell immediately after cell division, which has at t=0 the cell size $n_{0}$. One can define the probability $p_{n}^{division}$ for a cell of size *n* to divide,

(1) $$p_{n}^{division}=p=\frac{dn}{dn+kn}=\frac{1}{1+\frac{k}{d}}$$

One might notice that, in this stochastic model, the probability of division is independent of the current size of the cell. The probability to divide after adding exactly the size *n* can be estimated then as

(2) $$Q_{n}^{division}=p{\left(1-p\right)}^{n}\text{.}$$

The physical interpretation of this result is straightforward: no cell divisions occur during the first *n* events (only cell growth takes place with the probability 1−p at every step), whereas the (n+1)-th event triggers division with the probability *p*. One can now evaluate the average length added after the last division,

(3) $$\left\langle l\right\rangle =\sum_{n=0}^{\infty }nQ_{n}^{division}=\frac{1-p}{p}=\frac{k}{d}\text{.}$$

This is an important result, as it shows the same added length between divisions independently of the starting size of the cell. This picture corresponds to the adder scenario and the stochastic approach can explain it. The surprising observation here is that, despite the overall system being stochastic, the average added length between divisions is always constant, which is a deterministic result. The coupling between growth and division leads to a single-length scale determined by the ratio of corresponding rate constants. Thus, the stochastic-coupling framework not only recovers the experimentally observed adder dynamics but also provides microscopic arguments to explain this behavior.

To understand better the dynamics of cell growth and division, although at very qualitative level, let us consider a simple heuristic approximate description of the system (25). One can define n(t) as the average size of the cell at time *t*, and at t=0 the average cell size is $n\left(t=0\right)=n_{0}$. The following master equation might be written to describe the dynamics of average cell length (25):

(4) $$\frac{dn\left(t\right)}{dt}=kn\left(t\right)-2dn\left(t\right)\frac{n\left(t\right)}{2}\text{.}$$

The physical meaning of this heuristic expression is the following. The first term describes bacterial cell growth, which is proportional to the average cell length, and the second term approximately corresponds to changes in the average cell length due to division. The rate of division is dn(t), and the coefficient 2 reflects the creation of two new daughter cells from a single mother cell, whereas n(t)/2 corresponds to the decrease in the average cell length after division: from the cell of size *n*, two new cells of size n/2 are created. This equation can be easily solved at all times, producing

(5) $$n\left(t\right)=\frac{cke^{kt}}{1+dce^{kt}}\text{,}$$

where

(6) $$c=\frac{n_{0}}{k-dn_{0}}\text{.}$$

At large times, in this approach, the system will reach steady-state conditions with the stationary cell length given by

(7) $$n\left(t\to \infty \right)\equiv n_{st}=\frac{k}{d}\text{.}$$

This result again implies that there is one length scale in this system given by the ratio of growth and division rate constants, reflecting the coupling between the corresponding stochastic processes. The temporal evolution of the average length scale (see Eqn. (5)) can be made dimensionless (25),

(8) $$\frac{n\left(t\right)}{n_{st}}=\frac{e^{kt}}{\frac{n_{st}}{n_{0}}-1+e^{kt}}\text{.}$$

**Figure 2:**
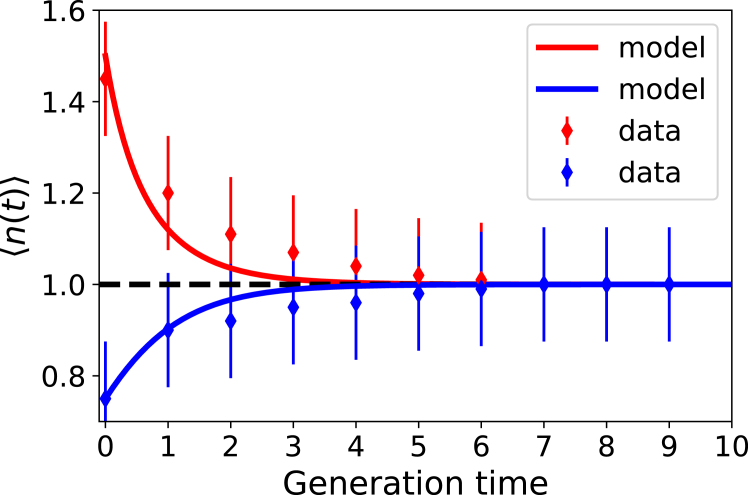
Comparison of theoretical predictions and experimental observations for normalized cell length in *E. coli* bacteria. Experimental data are from Ref. (17). The parameters k=0.01$\min^{-1}$ and d=0.1$\min^{-1}$ have been used in the analysis. The error bar for each symbol is defined as the standard error. The figure is reproduced with permission from Ref. (25).

Using experimental estimates for the parameters (17), k∼0.01 and d∼0.1 $\min^{-1}$, one could approximately evaluate $n_{st}∼10$. This expression has been used to describe the experimental observations for *E. coli* bacteria, as presented in Fig. 2 (25). As one can see, the stochastic-coupling approach faithfully reproduces the dynamics of length variations in bacteria. It also shows the robustness of the cell-size-regulation mechanism. Even for the newborn cells that deviate up to 50% from the average cell length, it only takes two or three divisions to return to the stationary limit (see Fig. 2).

It is possible to generalize this approach to more realistic descriptions of biological systems where many stochastic processes need to be included. One could suggest that, in this case, there are still a few more relevant stochastic processes that are orthogonal to each other, and coupling of those processes will again lead to specific spatial length scales. The role of specific biological regulation is to couple such stochastic processes and control their transition rates to tune the emerging length scales. This is a possible microscopic picture of how random processes can lead to spatial precision in biological systems.

## Temporal precision in bacterial cell lysis

**Figure 3:**
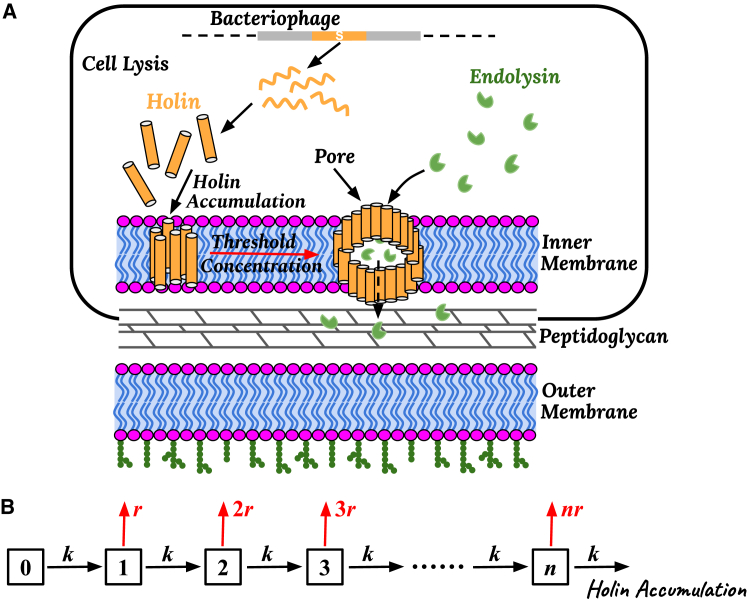
Stochastic model of phage-induced bacterial cell lysis. (*A*) Schematic diagram of a gram-negative bacterial cell infected by phage viruses. Holin proteins produced by the phage diffuse to the inner membrane, where they create pores after reaching a threshold concentration. Endolysin enzymes, also produced by the phage, pass through these pores and degrade the peptidoglycan layer of the cell wall. (*B*) Simplified discrete-state stochastic model of cell lysis, with states representing the number of holin molecules in the membrane. Holin accumulation occurs at a rate *k*, whereas cell lysis proceeds with a rate constant *r*. (*A*) Reprinted with permission from Ref. (26). Copyright 2024 Biophysical Journal.

Living systems are capable of achieving a high precision not only in spatial dimension but also in the dynamic aspects of underlying phenomena. Temporal precision in biological systems refers to the remarkable ability of various processes to occur at specific, predictable times, despite the random biochemical and environmental fluctuations within living cells. Often, this precision is achieved by the controlled accumulation of regulatory proteins, which reach specific threshold concentrations to trigger events. Despite strong advances in understanding the microscopic origin of biological noise (14,39,40,41,42), how cells can achieve such precise timing remains an open question. One well-studied example of precise temporal control is the process of cell lysis by *λ* bacteriophages, where the virus infects the bacterial cell and stimulates the production of specific proteins by the host, which, after reaching a critical concentration threshold in the cellular membrane, initiates cell lysis at precise times (43). These events are schematically shown in Fig. 3 *A*.

In *λ*-phage infection, the virus first injects its DNA into a bacterial cell and then follows a pathway of viral reproduction (44). During the late stages of this pathway, the phage prompts the host cell to synthesize holins, which are small, membrane-associated proteins, encoded by the *S* gene of the phage (45). These proteins accumulate in the inner membrane of bacterial cells (see Fig. 3 *A*). For a significant period, as shown experimentally, holins do not affect membrane integrity or any cell function (46), despite constantly increasing their concentration. Then, once the holin concentration reaches a precise threshold, a sudden, dramatic shift occurs: holins form large lesions (>300 nm) in the membrane (47), allowing phage-encoded endolysins (another important enzyme species) to access the cell wall and rapidly degrade it (46). This leads to swift cell lysis and the release of viral progeny. In gram-negative bacterial hosts, the cell lysis process also includes a secondary stage where spanin proteins fuse the inner and outer membranes, completing the cell opening and facilitating viral release (13,22,48,49,50).

The temporal precision of cell lysis by *λ* bacteriophage makes it a valuable model for studying precise timing events in biological systems, particularly as it has been extensively characterized with a wealth of quantitative experimental data (46,47,50,51,52). Multiple experimental observations of cell-lysis phenomena have already been obtained. For example, it was found that compounds known as “energy poisons” partially depolarize the cell membrane, and this can prematurely induce holin-triggered lysis, even at sub-threshold concentrations of holin proteins (46). Additionally, mutations in the holin gene (S105 allele) reveal sensitivity in lysis timing, especially in the three transmembrane domains crucial for holin function (53,54,55,56,57). Site-directed mutagenesis studies have shown that even single-amino-acid changes can significantly impact lysis timing, introducing increased variability relative to the wild-type (WT) *λ* phage, which exhibits minimal timing noise (15). This means that the distributions of cell lysis times are the narrowest for WT holin proteins. Surprisingly, some mutants achieve faster lysis than the WT, although with increased timing variability (15).

Several theoretical approaches have attempted to model cell lysis timing (14,15,58,59,60,61,62), treating it as a stochastic event tied to gene expression levels. The first-passage approach, for instance, can describe the stochastic process of regulatory proteins reaching threshold levels (14,59). However, all these methods face a serious limitation because they rely on phenomenological assumptions about threshold behavior without addressing the underlying molecular origins. In other words, the experimentally observed threshold must be a result of some biochemical and biophysical processes. Consequently, these theoretical approaches cannot fully explain the molecular mechanisms governing the precise timing of cell lysis (14,58,61). As a result, quantitative models could not connect the biophysical and biochemical details of holin accumulation and lysis timing at a molecular level, highlighting the need for further theoretical exploration to bridge these gaps.

To resolve these problems, a novel stochastic-coupling method has been proposed recently (26). It relies on the idea that almost deterministic dynamic behavior in biological systems might be achieved by coupling specific stochastic processes. This is very similar to the proposed mechanism of achieving spatial precision in cell-size regulation, as was discussed above. The difference is that, instead of spatial dimensions, this leads to narrow distribution of regulatory proteins, effectively mimicking thresholds, which eventually produces narrow distributions of cell lysis times.

To be more specific, the minimal stochastic model of cell lysis, as illustrated in Fig. 3 *B*, has been introduced for the analysis of cell-lysis phenomena (26). In this approach, the system is described as a set of discrete states, labeled *n*, where *n* is the number of holin proteins already translocated to the cellular membrane from the cytosol. Holin accumulation in the membrane is modeled with an effective rate *k*, which accounts for the holin movement to the cytoplasmic membrane, for the entry, and for the distribution within the membrane (26). Importantly, it is assumed that holins are stable proteins that do not degrade over the relevant timescales, which is consistent with experimental findings (63,64). The lysis (membrane breaking) in this model can occur from any state with the rate constant *r* (i.e., the rate per one holin protein) and it is proportional to the number of holins already present in the membrane. The assumption that the lysis rate scales with the number of holins is based on more microscopic arguments that holins must aggregate to form lesions in the membrane. This aggregation can be viewed as a chemical-reaction-like process where holin monomers assemble into functional oligomers, and thus the probability of membrane disruption is logically proportional to the number of holins. However, it was also argued that more complex power-law dependence of cell lysis rates (rate $∼n^{\alpha }$ with α>1) do not change the physics of this process, and, thus, the simplest linear dependence was assumed (26).

It is crucial to note here that the stochastic-coupling approach does not assume any thresholds. On the contrary, the threshold-like behavior naturally comes from the analysis of this theoretical model. To see this, one can introduce a probability for the system to observe the cell lysis from the state *n*,

(9) $$p_{n}^{lysis}=\frac{nr}{nr+k}=\frac{n}{n+x}\text{,}$$

where x=k/r is a coupling parameter. Another important quantity is the probability that the cellular membrane breaks for the first time (assuming that the system started in the state n=0 at t=0) after *exactly n* holin proteins enter the membrane,

(10) $$Q_{n}^{lysis}=\left(\prod_{j=1}^{n-1}\left(1-p_{j}^{lysis}\right)\right).p_{n}^{lysis}$$

**Figure 4:**
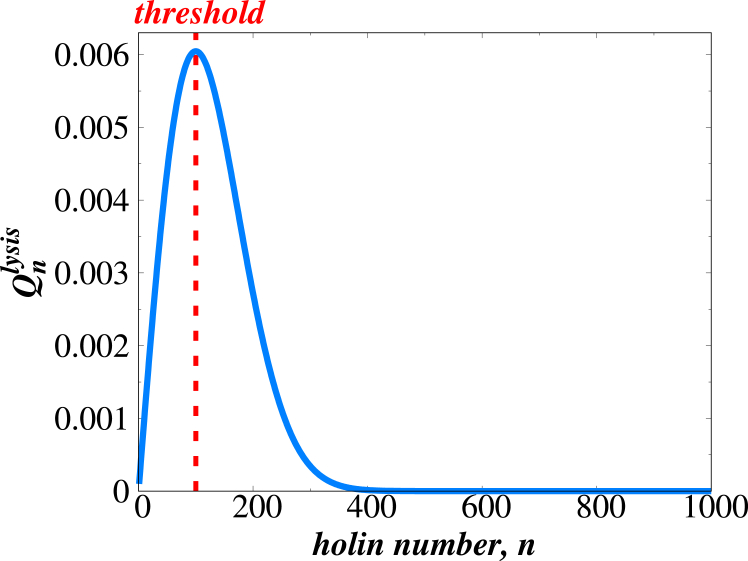
Theoretical prediction of the precise distribution of holin proteins triggering cell lysis. Calculations are performed using Eq. 10 with $x=10^{4}$.

Fig. 4 shows this quantity for realistic parameters of the *λ*-phage system. One can see that, although cell lysis can occur for different numbers of holin proteins in the membrane, there is a value $n_{\max}$ where this probability is maximal. It can be also shown that (26)

(11) $$n_{\max}≃x\text{,}$$

which implies that the balance between holin accumulation and holin removal due to cell lysis determines this important scale in the system. One can see that this mimics the effective threshold behavior as observed in experiments (15), and the maximal value $n_{\max}$ can be associated with the experimentally observed threshold. Thus, the stochastic-coupling method explains the appearance of the threshold without any phenomenological assumptions. It is the result of balancing between two stochastic processes in the system: the holin accumulation and holin removal due to cell lysis (26).

Another advantage of the stochastic-coupling model is that it allows one to explicitly estimate all dynamic properties of the system. For this purpose, one can explore a method of first-passage that has been successfully utilized in analysis of various chemical, biological, and physical phenomena (65,66). More specifically, one can define a function $F_{n}\left(m,t\right)$ as the probability density for cell lysis to occur for the first time in the state *m* (*m* holins in the membrane) if at t=0 the system started in the state *n* (*n* holins in the membrane). The temporal evolution of these first-passage probabilities is controlled by a set of backward master equations (26),

(12) $$\frac{dF_{n}\left(m,t\right)}{dt}=kF_{n+1}\left(m,t\right)-\left(k+nr\right)F_{n}\left(m,t\right)\text{,}$$

for 1≤n<m. In addition, at n=0, we have

(13) $$\frac{dF_{0}\left(m,t\right)}{dt}=kF_{1}\left(m,t\right)-kF_{0}\left(m,t\right)\text{,}$$

whereas for n=m it leads to

(14) $$\frac{dF_{m}\left(m,t\right)}{dt}=mrF_{cl}\left(m,t\right)-\left(k+mr\right)F_{m}\left(m,t\right)\text{,}$$

where $F_{cl}\left(m,t\right)$ is the probability of being found immediately after cell lysis. These equations can be solved explicitly using Laplace transformations, eventually producing in the realistic limit x≫1 the following distribution of cell lysis times (26):

(15) $$F_{0}\left(m,t\right)∼\frac{r{\left(kt\right)}^{m}e^{-kt}}{\left(m-1\right)!}\text{.}$$

This result can be intuitively explained using the following arguments. There are *m* fast events (x≫1) of adding the proteins into the membrane, and then cell lysis might occur with the rate *r* from the state *m*, but the system can also add another protein to the membrane with the rate *k*.

To fit experimental data, this function must be normalized because the overall probability for cell lysis to occur exactly from the state *m* is equal to m/x (26), leading to

(16) $$F_{0}\left(t\right)\equiv F_{0}\left(m,t,normalized\right)=\frac{k{\left(kt\right)}^{m}e^{-kt}}{m!}\text{.}$$

**Figure 5:**
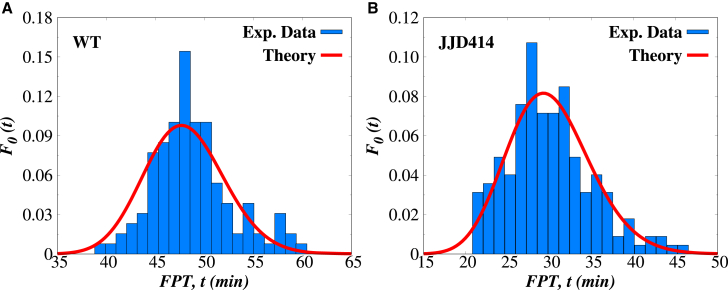
Theoretical fits of lysis time distributions for WT and mutant holins Theoretical fits of experimental cell lysis times distributions for (*A*) WT holin proteins and (*B*) JJD414 mutant holin species. Experimental data are from Ref. (15). In this analysis, we used m=⟨n⟩. The figure is reproduced with permission from Ref. (26).

These theoretical predictions have been used to describe experimental observations for cell lysis time distributions, as illustrated in Fig. 5. Analytical results have been used for optimal fitting of experimental data for both WT holins and mutant holin species, providing the estimates for kinetic rates and clarifying some important aspects of molecular mechanisms that govern the cell-lysis phenomena (26).

**Figure 6:**
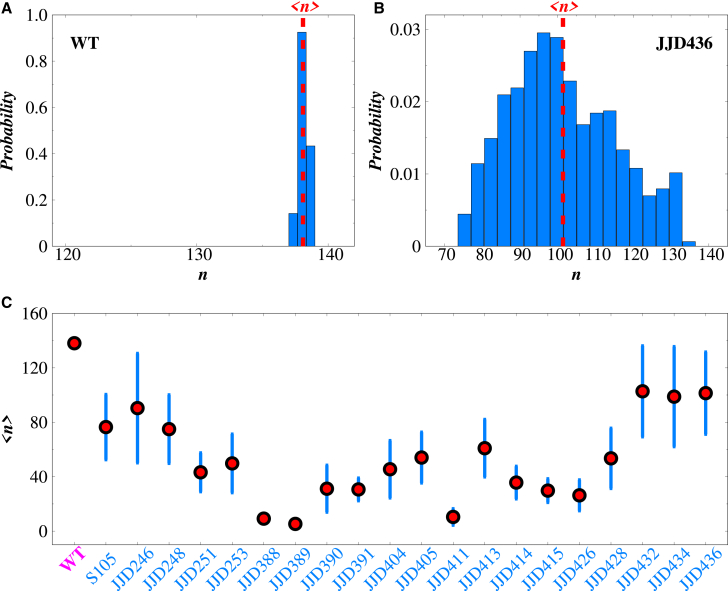
Spatial distribution and quantification of holin proteins in WT and mutant systems (*A*) Spatial distribution of holin protein in WT system. (*B*) Spatial distribution of the mutant JJD436 holin species. (*C*) Variation in the average number of holin proteins that stimulate cell lysis for WT and different mutant systems. Error bars are 95% confidence intervals, obtained after bootstrapping of 1000 replicas.The figure is reproduced with permission from Ref. (26).

The stochastic-coupling method is also helpful for better understanding the microscopic details that lead to precise timing, as illustrated in Fig. 6 (26). It has been argued that, for holin proteins to achieve precise timing, two conditions must be satisfied. The first one is that the number of holins in the membrane should be as large as possible, and the second one is that the spatial distribution should be narrow. As one can see in Fig. 6, WT holins satisfy both conditions, whereas the mutant species have either a broad spatial distribution or a smaller number of proteins inside the membrane.

It is important to note that the stochastic approach predicts a distribution of thresholds (see Fig. 4), which agrees with experimental observations (see Figs. 4 and 5). So, it is not a single specific threshold that specifies the triggering event in cell lysis. However, these distributions are still much more narrow than one would expect from a naive application of stochasticity in this system. This means that the cell lysis does not happen with comparable probability at all times, but rather there is a specific narrow range of times when this can occur with the highest probability.

One should also mention that, although we discuss spatial regulation here, our calculations explicitly evaluate the distributions of the numbers of holin proteins in the membrane. It raises a question of where the spatial aspect is. To answer this question, we notice that our theoretical approach estimates the protein distributions that lead to cell lysis for a fixed size of the inner membrane. Thus, if the size of the membrane is increased, a larger number of holin proteins will be needed. This is because the critical step in cell lysis is a protein aggregation that can be viewed as a chemical reaction that depends on the concentration of free proteins in the membrane. Thus, the number of proteins must correlate with the size of the membrane, connecting our theoretical analysis with spatial regulation arguments.

## Critical evaluation and future directions

In this review, we described a recently proposed stochastic theoretical framework to explain the molecular mechanisms of unexpectedly high precision in biological processes. The method relies on the coupling of several stochastic processes that result in supporting only narrow ranges of regulatory proteins in living systems. This might lead to spatial precision if the amount of these proteins correlates with the dimensions, as shown in the analysis of cell-size regulation. It might also lead to temporal precision if these proteins trigger specific events, as shown in the analysis of cell-lysis phenomena. The stochastic-coupling approach allows one to explicit estimate of all dynamic properties of the system, and excellent agreement with available experimental observations is observed in all cases. However, most importantly, the stochastic framework provides a plausible physical picture of how biological systems might control and regulate noisy biochemical and biophysical phenomena to deliver their precise functioning. It implicitly assumes that nature optimized the rates of underlying biochemical processes to achieve the targeted outcomes.

At the same time, despite the apparent success of the stochastic-coupling method in analyzing several biological phenomena, it is important to discuss its limitations. Only the simplest minimal stochastic models that coupled just two processes have been considered so far. This means that multiple biochemical and biophysical processes have been lumped together to achieve the simplest analytical description. It remains unclear how a more realistic picture of biological processes with many processes might affect the proposed mechanisms of precision. For example, will it lead to multiple length scales or not, and will the narrow distributions of regulatory proteins still be supported? In addition, theoretical analysis always assumed that these systems quickly reach stationary dynamics. However it is frequently unclear in biological phenomena whether transient processes are relevant or not. In addition, this theoretical framework implicitly assumes that the timescales of creating regulatory proteins are much shorter than the timescales of processes that support spatial and temporal precision. Although this is probably a reasonable approach for typical cellular conditions, it might fail under extreme situations (for example, when the bacterial cells are under low-food conditions or they actively fight virus infections). Another important aspect of biological processes that is neglected by this approach is the existence of feedback regulation. Furthermore, this theoretical method cannot explain what specific atoms or specific physical-chemical properties of relevant biological molecules are the most crucial for supporting precision. This can only be accomplished by more advanced atomistic computer simulations and machine-learning analysis, as was recently shown (67).

However, despite these limitations, the proposed stochastic-coupling method might be considered a powerful tool for investigating complex biological processes. This is because it gives simple physical explanations of molecular transformations in biological systems, which are supported by exact quantitative estimates of different properties and by excellent agreement with experimental observations. The stochastic method also stimulates new investigations by proposing quantitative predictions. It seems reasonable in future studies to couple this approach with advanced theoretical methods such as high-quality atomistic computer simulations and machine-learning analysis to achieve a better understanding of complex microscopic mechanisms in living systems.

## Acknowledgments

The work was supported by the 10.13039/100000928Welch Foundation (C-1559) and by the 10.13039/100009159Center for Theoretical Biological Physics sponsored by the 10.13039/100000001NSF (PHY-2019745).

## Author contributions

A.M. and A.B.K. designed the research, analyzed the data, and wrote the paper. A.M. performed the research.

## Declaration of interests

The authors declare no competing interests.
